# Prognostic variables and scores identifying the last year of life in COPD: a systematic review protocol

**DOI:** 10.1136/bmjopen-2016-011677

**Published:** 2016-09-15

**Authors:** Laura-Jane E Smith, Ifrah Ali, Patrick Stone, Liam Smeeth, Jennifer K Quint

**Affiliations:** 1 Department of Respiratory Epidemiology, Occupational Medicine and Public Health, Imperial College London, London, UK; 2 Imperial College School of Medicine, London, UK; 3 Marie Curie Palliative Care Research Unit, University College London, London, UK; 4 Department of Epidemiology and Population Health, London School of Hygiene and Tropical Medicine, London, UK

## Abstract

**Introduction:**

People living with advanced chronic obstructive pulmonary disease (COPD) suffer from significant morbidity, reduced quality of life and high mortality, and are likely to benefit from many aspects of a palliative care approach. Prognostic estimates are a meaningful part of decision-making and better evidence for such estimates would facilitate advance care planning. We aim to provide quality evidence on known prognostic variables and scores which predict a prognosis in COPD of <12 months for use in the community.

**Methods and analysis:**

We will conduct a systematic review of randomised or quasi-randomised controlled trials, prospective and retrospective longitudinal cohort and case–control studies on prognostic variables, multivariate scores or models for COPD. The search will cover the period up to April 2016. Study selection will follow the Preferred Reporting Items for Systematic Reviews and Meta-Analyses (PRISMA) guidelines, with data extraction using fields from the Critical Appraisal and Data Extraction for Systematic Reviews of Prediction Modelling Studies (CHARMS) checklist for multivariate models, and study quality will be assessed using a modified version of the Quality In Prognosis Studies (QUIPS) tool.

**Ethics and dissemination:**

The results will be disseminated through peer-reviewed publications and national and international conference presentations.

**Systematic review registration number:**

CRD42016033866.

**Strengths and limitations of this study**

- Broad search strategy planned in order to identify a range of individual prognostic variables and multidimensional scores.
- A focus on prognostic variables available in clinical practice (rather than, eg, genomics), such that the results will be meaningful to current practice.
- The use of validated protocols and tools for data extraction, risk of bias assessment and reporting.

- Search restricted to patients with relatively stable disease, so we are unable to comment on prognostic variables of most use during or immediately after an exacerbation.
- Despite rigorous use of protocols, there is a subjective element to any quality or risk of bias assessment.

## Introduction

Chronic obstructive pulmonary disease (COPD) is a complex, heterogeneous collection of conditions characterised by progressive irreversible expiratory airflow limitation. The prevalence of COPD is increasing globally and it is projected to be not only the third leading cause of death but also the seventh leading cause of disability-adjusted life years lost worldwide by 2030, representing an important public health challenge.^1^ Patients with advanced COPD have significant morbidity, reduced quality of life and high mortality.^2–6^

Despite national^7^ and international^8^ guidelines recommending a palliative care approach in severe COPD, patients are unlikely to access specialist services or elements of ‘general’ palliative care such as advance care planning, promotion of physical and psychosocial health and family or carer support.^6^ ^9–12^ Systematic identification of patients approaching the ‘end-of-life’ is a key recommendation of the end-of-life care strategy.^13^ The unpredictable disease trajectory of COPD^14^ makes this difficult. Policy literature uses the last year of life as the measure of those who are approaching death, and states that identification of this group is the first step in any palliative care process. However, there is no ‘gold standard’ method for predicting prognosis in COPD and no clear guidance on how to identify those in the last year of life. Easily measurable physiological parameters do not correlate well with mortality for individuals.^15^ There are alternative methods for identifying patients who may benefit from specific services, such as needs-based assessment.^16^ However, there are growing calls from patients, healthcare professionals and policymakers for better tools to aid prognostication which they see as a meaningful part of decision-making.^17^ ^18^ Clinician predictions of survival are often inaccurate, and improvement in accuracy of prognostic tools has been identified as a research priority.^19^

A number of variables have been identified which are useful in making predictions about prognosis in COPD, in addition to the degree of airflow obstruction which was the historical way of staging the disease.^20^ Scores that combine a number of variables have also been developed, in recognition of the fact that COPD is a multisystem disease. None of these scores are in widespread routine clinical use. This is partly because some variables used in these scores are not captured during routine care. The most well-validated prognostic score in COPD is the BODE (body mass index (BMI), FEV1% (forced expiratory volume in 1 s, % predicted for age and sex), MRC dyspnoea (Medical Research Council dyspnoea score) and 6MWT (6 min walk test)) index.^21^ However, this has significant limitations as it requires a 6MWT, not routinely performed or recorded in primary care. A modification of the BODE score, the ADO^22^ (age, MRC dyspnoea, FEV1%), has been developed to address this problem. These scores were developed in small cohorts, although efforts to modify and validate them in larger cohorts and different settings have demonstrated some external validity.^23–25^

A systematic review of multidimensional prognostic indices in COPD searched the literature up to September 2010.^26^ This study will have some important differences: we will consider the strength and utility not only of composite scores but also of individual prognostic variables. The only outcome of interest will be mortality, and not exacerbation or hospitalisation; and our focus will specifically be on prediction of prognosis towards the end of life (<12 months).

## Methods and analysis

### Aim

We aim to investigate known prognostic variables and scores that predict prognosis in COPD. We are specifically interested in those variables that contribute to risk assessment of patients in the community (ie, not hospitalised) for death within <12 months. In developing this protocol, we referred to the Preferred Reporting Items for Systematic Reviews and Meta-Analyses protocols (PRISMA-P) 2015 statement,^27^ a guide for the standard reporting of systematic review protocols.

### Inclusion criteria (participants, interventions, comparisons and outcomes)

Participants: Adults ≥35 years old with COPD as defined by GOLD.^1^

Interventions: We will include all randomised or quasi-randomised controlled trials, and prospective and retrospective longitudinal cohort and case–control studies which investigate prognostic variables, multivariate scores or models for COPD. We will include studies that describe the development, validation or impact assessment of prediction models.

Comparisons: Comparators and controls are less relevant in prognostic than intervention studies and may be absent in cohort studies.

Outcomes: The primary outcome of interest will be all-cause mortality ≤12 months following recording of prognostic variable or score.

### Exclusion criteria

We will exclude the following literature: abstracts only (eg, conference paper), case studies and reviews; studies that are on patients with α-1-antitrypsin deficiency, or those who have undergone lung transplantation, lung volume reduction surgery or comparative interventional bronchoscopic procedures; studies in which the diagnostic criteria for COPD is unclear or does not meet GOLD criteria; studies in which people with COPD form a subgroup and no separate reporting is available; studies requiring hospitalisation to acquire or measure the prognostic variable or score; studies examining short-term prognosis following an exacerbation or hospitalisation; studies that investigate prognostic markers not easily available in clinical practice (eg, biomarkers in development, invasive investigations); and studies in which the only exposure is occupational or environmental (eg, air pollution).

### Literature search

**Figure 1:**
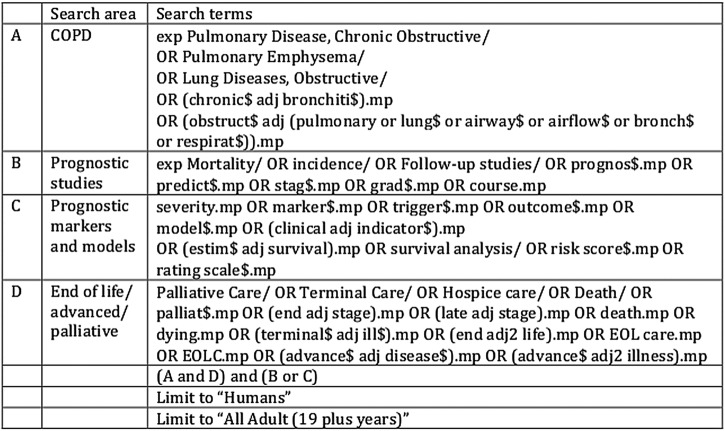
Example Ovid search strategy, developed with the help of a medical librarian.

We will search Ovid MEDLINE, EMBASE, the Cochrane database, Cochrane CENTRAL, DARE and CINAHL up to 30 April 2016. We will use medical subject heading and text words related to COPD, and broad strategies to identify prognostic studies and prognostic markers, focused on advanced disease and the end of life (see figure 1). Recognising potential limitations of electronic search strategies, we will supplement our search to identify potentially relevant studies from other sources, including reference lists of included studies, index-related articles on PubMed, and existing relevant reviews as well as Google Scholar search and ProQuest. Where necessary, authors will be contacted directly.

### Selection of studies and extraction of data

Two authors will scan the titles and abstracts of all literature retrieved by the initial search against inclusion and exclusion criteria and select articles for full-text review. All data will be downloaded to Zotero^28^ for data management. Two authors will review the full-text articles to assess eligibility for inclusion in the report. Differences of opinion will be resolved by consensus, or by arbitration by a third reviewer. The authors will extract data independently using a prespecified data extraction tool. This will include details of the study setting, study design, population, diagnostic criteria for COPD (including cut-points for FEV1% predicted), method of measurement of each prognostic variable and outcome definition. In addition, it will include fields relevant to multivariate models based on the CHARMS checklist^29^ such as modelling method, handling of predictors, method for selection of predictors, shrinkage of predictor weights, univariate and multivariate associations, model performance and evaluation. This will be piloted on the first five full-text reviews to ensure standardised use of the tool. The process of literature selection and reasons for exclusion will be fully documented and a PRISMA^27^ flow diagram will be constructed.

### Quality assessment

Two reviewers will assess quality and risk of bias of eligible studies based on prespecified domains. We will use an approach based on the Quality In Prognosis Studies (QUIPS) tool,^30^ specifically designed for prognostic reviews. We will consider questions under six domains: study participation and attrition, prognostic factor measurement, outcome measurement, confounding measurement and account, analysis and other. Consensus will be reached by discussion, or by arbitration by a third reviewer.

### Data synthesis

Owing to clinical and methodological heterogeneity in potentially included studies identified in the scoping review, it is not expected that formal meta-analysis will be possible. The planned method for evidence synthesis is therefore a narrative synthesis of all identified evidence. We will summarise the range of outcome predictors that have been studied to date. With regard to composite scores, we will assess not only the quality of model building but also the degree to which the scores have been externally validated and to what degree clinical utility and impact has been assessed.

We anticipate that many of the studies will be in restricted populations, such as trial populations, that may not represent the population of patients with COPD in the community. We will thus be cognisant of and comment on possible spectrum bias^31^ and the implications for generalisability of findings. An assessment of the strength of evidence for each prognostic variable or score included will be formulated based on GRADE evidence profiles.^32^

## Ethics and dissemination

No ethical approval is required, since this study is a synthesis of published studies. The results will be submitted for peer-reviewed publication and will be presented at national and international conferences.

The protocol has been registered in the PROSPERO database: CRD42016033866. Any amendments to the study protocol will be documented contemporaneously on the PROSPERO database site.
